# Liraglutide Protects Cardiomyocytes against Isoprenaline-Induced Apoptosis in Experimental Takotsubo Syndrome

**DOI:** 10.3390/biomedicines12061207

**Published:** 2024-05-29

**Authors:** Zorislava Bajic, Tanja Sobot, Ljiljana Amidzic, Natasa Vojinovic, Sanja Jovicic, Milica Gajic Bojic, Dragan M. Djuric, Milos P. Stojiljkovic, Sergey Bolevich, Ranko Skrbic

**Affiliations:** 1 Department of Physiology, Faculty of Medicine, University of Banja Luka, 78 000 Banja Luka, Bosnia and Herzegovina; tanja.sobot@med.unibl.org; 2 Centre for Biomedical Research, Faculty of Medicine, University of Banja Luka, 78 000 Banja Luka, Bosnia and Herzegovina; ljiljana.amidzic@med.unibl.org (L.A.); natasa.vojinovic@med.unibl.org (N.V.); sanja.jovicic@med.unibl.org (S.J.); milica.gajic@med.unibl.org (M.G.B.); milos.stojiljkovic@med.unibl.org (M.P.S.); ranko.skrbic@med.unibl.org (R.S.); 3 Department of Biology of Cell and Human Genetics, Faculty of Medicine, University of Banja Luka, 78 000 Banja Luka, Bosnia and Herzegovina; 4 Department of Histology and Embryology, Faculty of Medicine, University of Banja Luka, 78 000 Banja Luka, Bosnia and Herzegovina; 5 Department of Pharmacology, Toxicology and Clinical Pharmacology, Faculty of Medicine, University of Banja Luka, 78 000 Banja Luka, Bosnia and Herzegovina; 6 Faculty of Medicine, Institute of Medical Physiology “Richard Burian”, University of Belgrade, 11 000 Belgrade, Serbia; dr_djuric@yahoo.com; 7 Department of Pathologic Physiology, First Moscow State Medical University I.M. Sechenov, 119435 Moscow, Russia; bolevich2011@yandex.ru

**Keywords:** isoprenaline, myocardial injury, Takotsubo syndrome, liraglutide, apoptosis, NFκ-B

## Abstract

Takotsubo syndrome (TTS) is a stress-induced cardiomyopathy, characterized by an increased concentration of catecholamines, free radicals, and inflammatory cytokines, endothelial dysfunction, and increased apoptotic activity. High doses of isoprenaline are used in animal models to induce Takotsubo (TT)-like myocardial injury. The aim of the study was to investigate the antiapoptotic effects of liraglutide in experimental TTS and its role in the NF-κB pathway. Wistar rats were pretreated with liraglutide for 10 days, and on days 9 and 10, TT-like myocardial injury was induced with isoprenaline. After the sacrifice on day 11, hearts were isolated for histopathological and immunohistochemical analysis. Liraglutide reduced isoprenaline-induced cardiomyocyte apoptosis by decreasing cleaved caspase-3 (CC3), BCL-2-associated X protein (BAX), and NF-κB and increasing B-cell lymphoma/leukemia-2 (BCL-2). An increase in NF-κB in isoprenaline-treated rats was in positive correlation with proapoptotic markers (BAX and CC3) and in negative correlation with antiapoptotic marker BCL-2. Liraglutide increased BCL-2 and decreased NF-κB, BAX, and CC3, preserving the same correlations of NF-κB to apoptotic markers. It is concluded that liraglutide protects cardiomyocytes against isoprenaline-induced apoptosis in experimental TT-like myocardial injury through downregulation of the NF-κB pathway.

## 1. Introduction

Takotsubo syndrome (TTS), also known as broken heart syndrome, apical ballooning syndrome, or ampulla cardiomyopathy, is a stress-induced cardiomyopathy that occurs after extreme physical or emotional stress [1,2]. The mortality rate of TTS in the hospital setting is similar to that of acute coronary syndrome. TTS occurs more often in women over the age of 60, but it has a worse prognosis in men. The symptoms and signs of this syndrome are like those of acute myocardial infarction [3,4], but the absence of coronary occlusion distinguishes it from the acute coronary syndrome [3]. TTS can cause serious complications, such as heart rhythm disorders and left ventricular outflow tract obstruction, with serious consequences [4]. The significantly elevated concentrations of adrenaline and noradrenaline in the acute phase of TTS compared to the acute myocardial infarction suggest that the main cause of this syndrome could be hyperactivity of the sympathetic nervous system [3].

Experimental studies have shown that the administration of high doses of adrenaline or isoprenaline in animal models causes acute reversible cardiac dysfunction like TTS [3]. The mechanism by which isoprenaline induces myocardial injury involves the production of highly cytotoxic free radicals via the autoxidation of catecholamines. These free radicals cause lipid peroxidation, oxidative stress, and inflammation, which can lead to the development of cardiomyopathy [5,6].

Glucagon-like peptide 1 (GLP-1) is an incretin hormone of the small intestine, secreted after a meal rich in carbohydrates, especially glucose. GLP-1 receptors are widely distributed in multiple organs, including the pancreas, gastrointestinal tract, central nervous system, and cardiovascular system. GLP-1 increases pancreatic insulin production, proliferation, and survival of beta-cells in the pancreas and reduces glucagon secretion [7]. Due to the beneficial effects of GLP-1, the idea of creating synthetic forms of GLP-1 receptor agonists (GLP-1RAs) was born. GLP-1RAs were created primarily for the management of type 2 diabetes mellitus (T2DM) [7]. Liraglutide is a GLP-1RA that, apart from its primary effect of lowering blood glucose levels, also has cardioprotective and neuroprotective effects [7,8,9,10]. Liraglutide improves endothelial and cardiac function through the suppression of oxidative stress and inflammation [8,9,10].

The aim of this study was to investigate the antiapoptotic effects of liraglutide in experimental TTS.

## 2. Materials and Methods

### 2.1. Experimental Animals

Male Wistar rats, weighing 180–220 g, were housed under controlled laboratory conditions: room temperature 21 ± 2 °C, air humidity 55 ± 5%, and 12/12 h light/dark cycle. The experimental animals, laboratory protocols, and experimental design were approved by the Ethics Committee for the Protection and Welfare of Experimental Animals of the Faculty of Medicine at the University of Banja Luka, The Republic of Srpska, Bosnia and Herzegovina (dated 1 June 2022, number 18/1.190-13/22). Animal housing was conducted according to the Guide for the Care and Use of Laboratory Animals of the National Institute for Health (NIH).

### 2.2. Experimental Design

Experimental animals were treated with liraglutide or saline for 10 days, and on days 9 and 10, myocardial injury was induced by isoprenaline. The experimental model of Takotsubo (TT)-like myocardial injury included the administration of two doses of isoprenaline (each 85 mg/kg) with 24 h intervals, as previously described [11,12]. Rats were divided into 4 groups: (1) control group (C) n = 6—rats were treated with saline 1 mL/kg sc for 10 days, and on days 9 and 10, they were treated with saline 1 mL/kg sc; (2) liraglutide group (L) n = 6—rats received liraglutide in a dose of 1.8 mg/kg sc from days 1 to 10 and were treated with saline 1 mL/kg sc on days 9 and 10; (3) isoprenaline group (I) n = 8—experimental animals were treated with saline 1 mL/kg sc for 10 days, and myocardial injury was induced by application of isoprenaline 85 mg/kg sc on days 9 and 10; (4) liraglutide + isoprenaline group (L + I group) n = 9—rats received liraglutide in a dose of 1.8 mg/kg sc for 10 days, and isoprenaline 85 mg/kg sc was administered on days 9 and 10.

At the end of the experiment, on the 11th day, all animals were sacrificed.

### 2.3. Histopathological Examination and Semiquantitative Analysis

To analyze the cardioprotective effects of liraglutide, 24 h after the last treatment, all rats were anesthetized with ketamine 90 mg/kg and xylazine 10 mg/kg ip and sacrificed. The Leica TP 1020 tissue processor was used to process tissue samples, which were embedded in paraffin blocks after 48 h of fixation in 4% formaldehyde. Using a Rotatory 3003 pfm microtome, each sample was cut to a thickness of 4 μm and stained with hematoxylin and eosin as a routine procedure. The obtained samples were analyzed with a binocular Leica DM 6000 microscope that was fitted with a Leica DFC310FX camera. A 5-point semiquantitative scale [13,14,15] was used to assess tissue damage on the entire visual field at 20× magnification.

Briefly, the tissue damage score was as follows: Score 1—normal findings, without morphologic changes; Score 2—single cell with granules of small size, slightly expanded, and normal nuclei; Score 3—more than 50% cells with mild cytoplasmic vacuolization and nucleoplasm and pycnotic nuclei, indicating overall mild damage; Score 4—all cells with pronounced vacuolization of cytoplasm and nucleoplasm and pycnotic nuclei, indicating moderate damage; Score 5—pronounced plasmolysis and karyolysis and diffuse infiltration of polymorphonuclear cells that surround phagocyte dead cells, bleeding, and interstitial edema, suggesting severe damage.

The LAS V4.12 software was used to perform a morphometric examination of tissue structures with a magnification of 40×. The number of examined visual fields (N) is determined according to the formula N = (20 × SD/X)2, where SD is the standard deviation, and X is the mean value of the results obtained in a pilot study on 20 visual fields [16]. Along with the thickness of the right ventricular wall, the diameter of cardiomyocytes present in the visual field was measured on a longitudinal section near the nucleus. Changes in cardiomyocyte diameter are associated with changes in the expression of proapoptotic and antiapoptotic markers, as well as DNA damage [17,18,19,20].

### 2.4. Analysis of Cardiomyocyte Apoptosis by DNA Fragmentation with TUNEL Method

To determine cell apoptosis in the myocardium, the terminal deoxynucleotidyl transferase-mediated (TUNEL) assay was used [21,22,23,24]. Paraffin sections of 4 µm thickness were used for this assay. Determination was made with an Abcam (Cambridge, UK) TUNEL Assay Kit—HRP-DAB (ab206386) according to the instructions of the manufacturer. The pathologist analyzed slides on a microscope Leica DM6000 B at a magnification of 400×. Slides were examined, and TUNEL-positive cells were counted in 10 non-successive fields in every sample. The apoptotic index (AI) was calculated as the percentage of apoptotic cardiomyocytes in the total number of cardiomyocytes [23]:

$$AI=\frac{number\;of\;TUNEL\;positive\;cardiomyocytes}{total\;number\;of\;cardiomyocytes}\cdot 100.$$

### 2.5. Analysis of Regulating Molecules of Apoptosis by Immunohistochemistry

Sections embedded in paraffin were immunostained with primary antibodies. For cleaved caspase-3 (CC3) detection in the cytoplasm, rabbit polyclonal anti-caspase-3 antibody (Asp 175) was used, and for BAX (BCL-2-associated X protein) detection in the cytoplasm, rabbit monoclonal anti-BAX antibody (ab32503, Abcam) was used. For cytoplasmic detection of BCL-2 (B-cell lymphoma/leukemia-2), the rabbit polyclonal anti-BCL-2 antibody (ab196495, Abcam) was used. For nuclear and cytoplasmic detection of the nuclear factor-kappa B (NF-κB), rabbit polyclonal anti-NF-κB p65 (ab16502, Abcam) was used.

Tissue sections were deparaffinized in xylene, then rehydrated in decreasing concentrations of ethyl alcohol, and the antigen was retrieved with a citric acid solution. For the reduction of nonspecific staining, slides were treated with 3% hydrogen peroxide for 10 min. Primary antibodies were used according to the manufacturer’s protocol and in appropriate dilution. After incubation and washing, the immunolabeling was detected using HRP Polymer (UltraVision LP Detection System, Thermo Scientific, Fremont, CA, USA) following the manufacturer’s specifications. 3,3’-Diaminobenzidine (DAB) was used as the chromogen. The slides were then counterstained with hematoxylin solution, dehydrated, and mounted. The Leica DM6000 B microscope (Wetzlar, Germany) at 400× magnification was used for slide analysis. Slides were examined, and immune-positive cells (cardiomyocytes with a characteristic optical expression of CC3, BAX, BCL-1, and NF-κB) were counted in 10 non-successive fields in every sample with a magnification of 400×. The percentage of immune-positive cardiomyocytes was calculated according to the formula [23]:

$$\%\;of\;immune-positive\;cardiomyocytes=\frac{number\;of\;positive\;cardiomyocytes}{total\;number\;of\;cardiomyocytes}\;\cdot 100$$

### 2.6. Statistical Analyses

Statistical analysis was performed with IBM-SPSS Statistics version 20.0 software (SPSS, Inc., Chicago, IL, USA). To evaluate the mean values, the analysis of variance (ANOVA) test was used. Post hoc analysis was conducted using the Bonferroni test. The results are presented as mean ± standard error, and *p* < 0.05 was considered statistically significant. For testing correlations, the Pearson test was used.

## 3. Results

### 3.1. Effects of Liraglutide on Morphological Features of Myocardium

**Figure 1:**
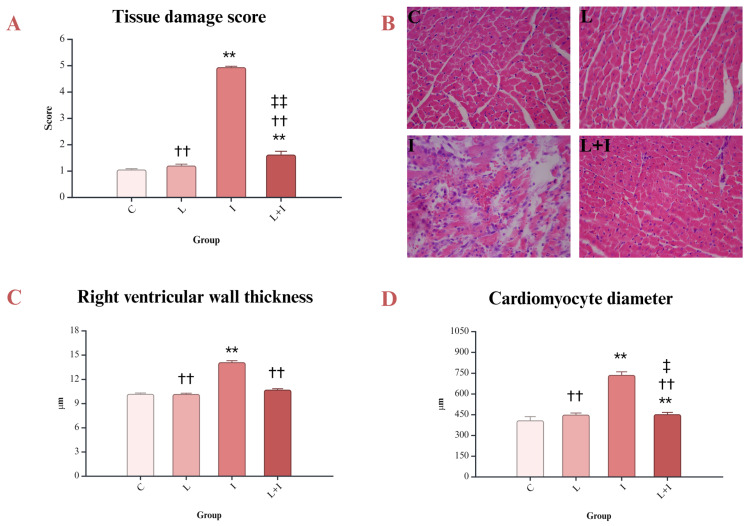
Morphological changes in myocardium. (**A**) Tissue damage score of the myocardium; (**B**) histological characteristics of the myocardium (H&E stain, magnification 20×); (**C**) right ventricular wall thickness; (**D**) cardiomyocyte diameter; control (C) group—saline 1 mL/kg sc for 10 days, and on days 9 and 10, saline 1 mL/kg sc; isoprenaline (I) group—saline 1 mL/kg sc for 10 days, and on days 9 and 10, isoprenaline 85 mg/kg sc; liraglutide (L) group—liraglutide 1.8 mg/kg sc for 10 days, and on days 9 and 10, saline 1 mL/kg sc; liraglutide + isoprenaline (L + I) group—liraglutide 1.8 mg/kg sc for 10 days, and on days 9 and 10, isoprenaline 85 mg/kg sc; ** *p* < 0.001 versus (vs.) C; †† *p* < 0.001 vs. I; ‡ *p* < 0.05 vs. L; ‡‡ *p* < 0.001 vs. L.

The study analyzed not only the morphological characteristics of the heart but also the thickness of the right ventricular wall and the diameter of cardiomyocytes. Myocardial morphology was severely damaged by isoprenaline. Histological examination showed that liraglutide prevented isoprenaline-induced damage (*p* < 0.001 I vs. L + I) (Figure 1A,B). Although histological analysis revealed that isoprenaline causes bleeding, inflammation, and interstitial edema, examination of cardiomyocytes showed that isoprenaline also induces an increase in cardiomyocyte diameter (Figure 1C). Appropriate magnification allowed visualization of the right ventricular wall, which revealed an increase in right ventricular wall thickness and an increase in cardiomyocyte diameter after isoprenaline administration. These findings indicate that increased thickness of the ventricular wall is not caused only by interstitial edema but also by cardiomyocyte hypertrophy. Liraglutide prevented these changes (*p* < 0.001 L + I vs. I) (Figure 1D).

### 3.2. Effects of Liraglutide on Apoptosis and Apoptotic Pathway

**Figure 2:**
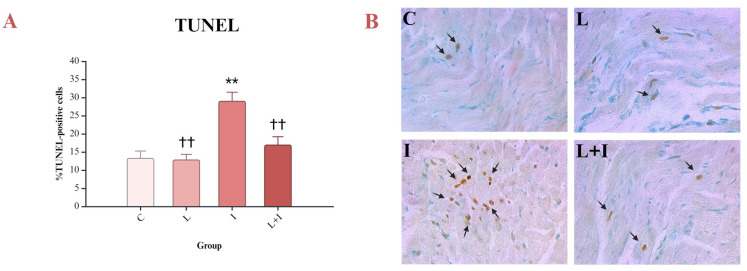
Effects of liraglutide on DNA fragmentation: (**A**) Percent of TUNEL-positive cells; (**B**) tissue sections of four different groups with arrows pointing TUNEL-positive cells; TUNEL—terminal deoxynucleotidyl transferase-mediated dUTP nick end labeling assay; control (C) group—saline 1 mL/kg sc for 10 days, and on days 9 and 10 saline, 1 mL/kg sc; isoprenaline (I) group—saline 1 mL/kg sc for 10 days, and on days 9 and 10, isoprenaline 85 mg/kg sc; liraglutide (L) group—liraglutide 1.8 mg/kg sc for 10 days, and on days 9 and 10, saline 1 mL/kg sc; liraglutide + isoprenaline (L + I) group—liraglutide 1.8 mg/kg sc for 10 days, and on days 9 and 10, isoprenaline 85 mg/kg sc; ** *p* < 0.001 vs. C; †† *p* < 0.001 vs. I.

The presence of apoptosis and the apoptosis pathway were analyzed by the TUNEL assay and immunohistochemical reaction of appropriate antibodies (anti-BAX, anti-CC3, anti-NF-κB, anti-BCL-2). Apoptosis is characterized by DNA fragmentation, which can be detected by the TUNEL assay. The percentage of TUNEL-positive cells was increased in the myocardium of isoprenaline-treated rats. Pretreatment with liraglutide prevented isoprenaline-induced nuclear apoptosis (L + I vs. I, *p* < 0.001) (Figure 2A,B).

**Figure 3:**
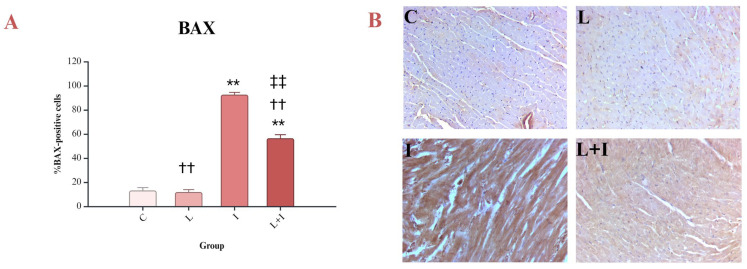
Effects of liraglutide on BAX: (**A**) Percent of BAX-positive cells; (**B**) tissue sections of four different groups; BAX—BCL-2-associated X protein; control (C) group—saline 1 mL/kg sc for 10 days, and on days 9 and 10, saline 1 mL/kg sc; isoprenaline (I) group—saline 1 mL/kg sc for 10 days, and on days 9 and 10, isoprenaline 85 mg/kg sc; liraglutide (L) group—liraglutide 1.8 mg/kg sc for 10 days, and on days 9 and 10, saline 1 mL/kg sc; liraglutide + isoprenaline (L + I) group—liraglutide 1.8 mg/kg sc for 10 days, and on days 9 and 10, isoprenaline 85 mg/kg sc; ** *p* < 0.001 vs. C; †† *p* < 0.001 vs. I; ‡‡ *p* < 0.001 vs. L.

The myocardial cells showed a strong immunohistochemical reaction when treated with an anti-BAX antibody. Liraglutide prevented isoprenaline-induced apoptosis by decreasing the percentage of BAX-positive cells (L + I vs. I, *p* < 0.001) (Figure 3A,B).

**Figure 4:**
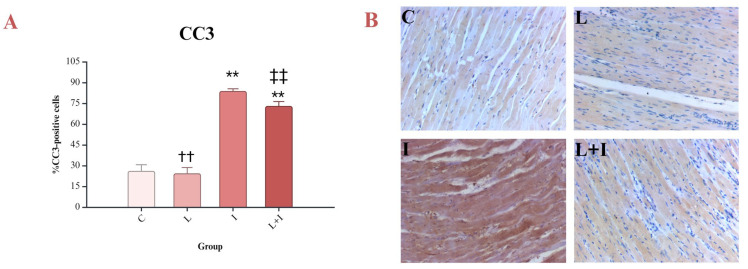
Effects of liraglutide on CC3: (**A**) Percent of CC3-positive cells; (**B**) immunohistochemical reaction of myocardium; CC3—cleaved caspase-3; control (C) group—saline 1 mL/kg sc for 10 days, and on days 9 and 10, saline 1 mL/kg sc; isoprenaline (I) group—saline 1 mL/kg sc for 10 days, and on days 9 and 10, isoprenaline 85 mg/kg sc; liraglutide (L) group—liraglutide 1.8 mg/kg sc for 10 days, and on days 9 and 10, saline 1 mL/kg sc; liraglutide + isoprenaline (L + I) group—liraglutide 1.8 mg/kg sc for 10 days, and on days 9 and 10, isoprenaline 85 mg/kg sc; ** *p* < 0.001 vs. C; †† *p* < 0.001 vs. I; ‡‡ *p* < 0.001 vs. L.

Isoprenaline-treated rats showed an increase in CC3-positive cardiomyocytes, indicating an increase in cardiac apoptosis. Liraglutide alleviated CC3-mediated apoptosis (L + I vs. I, *p* < 0.001) (Figure 4A,B).

**Figure 5:**
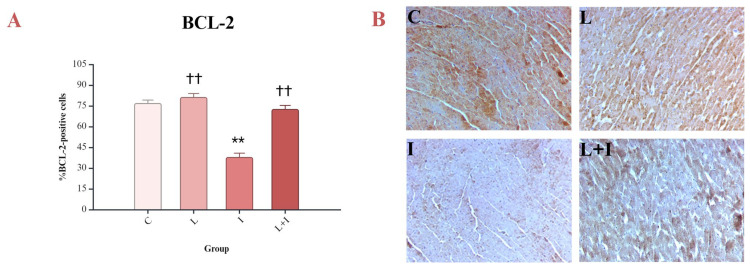
Effects of liraglutide on BCL-2: (**A**) Percent of BCL-2-positive cells; (**B**) immunohistochemical reaction of four different groups of experimental animals; BCL-2—B-cell lymphoma/leukemia-2; control (C) group—saline 1 mL/kg sc for 10 days, and on days 9 and 10, saline 1 mL/kg sc; isoprenaline (I) group—saline 1 mL/kg sc for 10 days, and on days 9 and 10, isoprenaline 85 mg/kg sc; liraglutide (L) group—liraglutide 1.8 mg/kg sc for 10 days, and on days 9 and 10, saline 1 mL/kg sc; liraglutide + isoprenaline (L + I) group—liraglutide 1.8 mg/kg sc for 10 days, and on days 9 and 10, isoprenaline 85 mg/kg sc; ** *p* < 0.001 vs. C; †† *p* < 0.001 vs. I.

The results showed that isoprenaline decreases the percentage of BCL-2-positive cells, and liraglutide manages to increase this antiapoptotic marker’s levels (L + I vs. I, *p* < 0.001) (Figure 5A,B).

**Figure 6:**
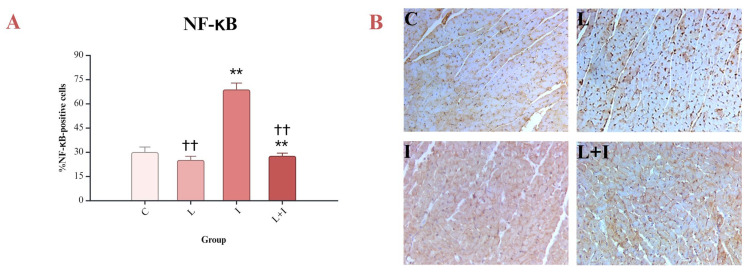
Effects of liraglutide on NF-κB: (**A**) Percent of NF-κB-positive cells; (**B**) immunohistochemical reaction of the myocardium; NF-κB—nuclear factor kappa B; C—control group (saline for 10 days and saline on days 9 and 10); I—isoprenaline group (saline for 10 days and isoprenaline 85 mg/kg on days 9 and 10); L—liraglutide group (liraglutide 1.8 mg/kg for 10 days and saline on days 9 and 10); L + I—liraglutide + isoprenaline group (liraglutide 1.8 mg/kg for 10 days and isoprenaline 85 mg/kg on days 9 and 10); ** *p* < 0.001 vs. C; †† *p* < 0.001 vs. I.

Testing the myocardium for NF-κB, one of the signal molecules that are involved in inflammation and oxidative stress pathways, showed a strong immunohistochemical reaction. Pretreatment with liraglutide decreased the percentage of NF-κB-positive cells (L + I vs. I, *p* < 0.001) (Figure 6A,B).

**Figure 7:**
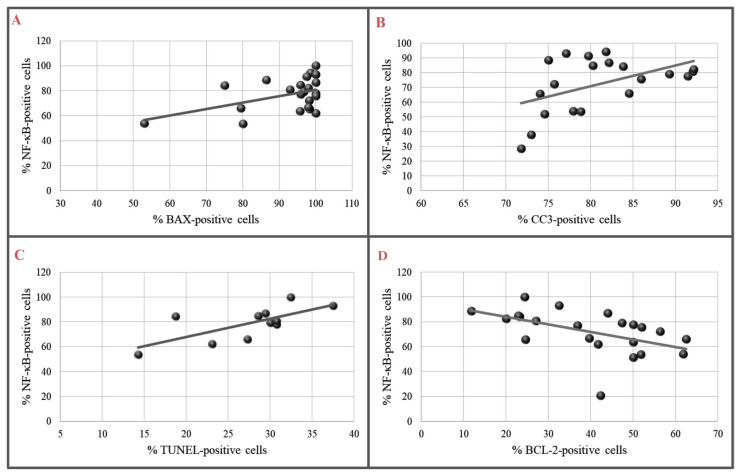
Correlation of NF-κB with proapoptotic and antiapoptotic markers in I group: (**A**) Correlation of NF-κB- and BAX-positive cells (Pearson correlation r = 0.461, *p* < 0.05); (**B**) correlation of NF-κB- and CC3-positive cells (Pearson correlation r = 0.489, *p* < 0.05); (**C**) correlation of NF-κB- and TUNEL-positive cells (Pearson correlation r = 0.710, *p* < 0.05); (**D**) correlation of NF-κB- and BCL-2-positive cells (Pearson correlation r = −0.507, *p* < 0.05).

Correlations between NF-κB and apoptotic markers, such as BAX, CC3, BCL-2, and DNA fragmentation, were analyzed in isoprenaline-induced TT-like myocardial injury. There were statistically significant positive correlations between NF-κB and proapoptotic markers (BAX, CC3) as well as DNA fragmentation. The correlation between NF-κB and the antiapoptotic marker (BCL-2) was negative with statistical significance (Figure 7A–D).

**Figure 8:**
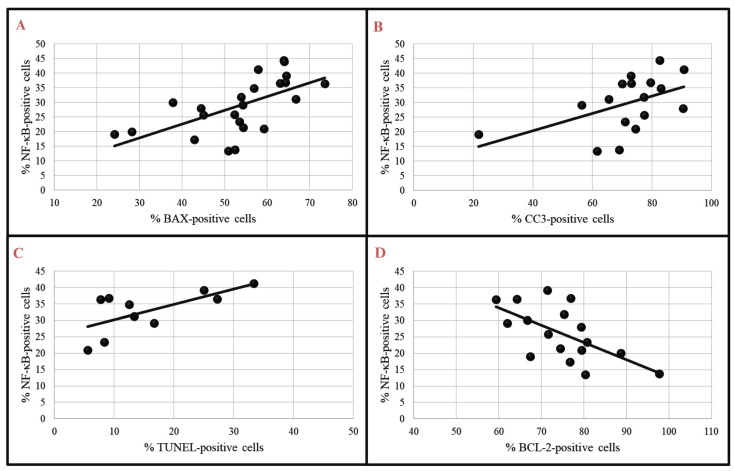
Correlation of NF-κB with proapoptotic and antiapoptotic markers in L + I group: (**A**) Correlation of Nf-κB- and BAX-positive cells (Pearson correlation r = 0.611, *p* < 0.05); (**B**) correlation of NF-κB- and CC3-positive cells (Pearson correlation r = 0.505, *p* < 0.05); (**C**) correlation of NF-κB- and TUNEL-positive cells (Pearson correlation r = 0.663, *p* < 0.05; (**D**) correlation of NF-κB- and BCL-2-positive cells (Pearson correlation r = −0.618, *p* < 0.05).

Correlations between NF-κB and proapoptotic or antiapoptotic markers were also analyzed in TT-like myocardial injury of rats pretreated with liraglutide. Correlations between NF-κB and proapoptotic markers were positive and statistically significant, similar to the I group, and the correlation between NF-κB and antiapoptotic marker, BCL-2, was similar to the I group, negative and statistically significant (Figure 8A–D).

## 4. Discussion

TTS is characterized by cardiac systolic dysfunction with characteristic apical ballooning and ECG changes similar to acute myocardial infarction, such as ST-segment elevation or T-wave inversion. The main difference between TTS and myocardial infarction, in addition to the absence of coronary occlusion, is the possibility of recovery from ventricular dysfunction and ECG changes [4]. The increased concentration of blood catecholamines in TTS can cause coronary microvascular dysfunction and spasms of epicardial coronary vessels [25,26,27,28,29]. Catecholamines can increase cardiac contraction via β1 and β2 myocardial receptors in physiological conditions [4]. Although the heart has both types of receptors, about 80% are β1, and only about 17% are β2. Also, their role can be different because stimulation of β1 can lead to increased cardiac contraction, while stimulation of β2 receptors can have stimulatory or inhibitory effects [30]. Therefore, catecholamine excess can cause a change in ventricular wall motion, in the sense of decreased motility of the apical segment and increased motility of the basal segment of the heart [4]. That change can be explained by the density and sensitivity of adrenoreceptors in different areas of the heart. In the apical segment of the heart, the density of β2/β1 receptors is higher than in the basal segment, and the sensitivity of adrenoreceptors to catecholamines in the apex is higher than on the base of the heart [28]. In a rat model of TTS, the inhibition of β1 adrenergic receptors alleviates akinesia, while the blockade of β2 receptors does not influence akinesia, suggesting an important role of β1 adrenergic receptors in the pathophysiological mechanism of TTS [4]. Administration of β-adrenergic agonists, such as isoprenaline, can induce clinical symptoms and signs of TTS [28]. An excess of catecholamines can induce oxidative stress and inflammation, suggesting their involvement in the pathophysiology of TTS [31].

The conducted study showed myocardial damage in the experimental model of TTS. The myocardium of isoprenaline-treated rats was characterized by inflammation, interstitial edema, and vacuolization of the cytoplasm. Similar histological findings in experimental TTS were presented by Kołodzińska et al. [32].

The results of the current study showed that liraglutide prevented these changes, showing its protective effects. The histological analysis included measurement of the right ventricular wall thickness. In rats treated with isoprenaline, the thickness of the right ventricular wall was increased. The question was whether the increased thickness of the right ventricular wall was due to interstitial edema or due to an increased diameter of cardiomyocytes. Thus, cardiomyocyte diameter was measured, and the results showed an increase in cardiomyocyte diameter. Cardiomyocyte hypertrophy was also confirmed in isoprenaline-induced TTS by other authors [32]. Liraglutide reduced the right ventricular wall thickness and cardiomyocyte hypertrophy in isoprenaline-treated rats. The change in shape and function of the heart after myocardial injury is caused by cardiomyocyte hypertrophy and deposition of collagen fibers [33]. Liraglutide reduces infiltration of macrophages, fibroblast differentiation, and collagen deposition and attenuates cardiac hypertrophy [33,34,35,36]. In our previous studies, we showed that the administration of isoprenaline in animal models of myocardial injury was associated with an increased level of ROS [15,37]. ROS can initiate the NF-κB pathway, and NK-κB can increase the activity of enzymes included in ROS generation, such as NADPH dehydrogenase, and reduce the activity of antioxidative enzymes involved in ROS degradation [38,39]. The results of the study showed a significant increase in myocardial NF-κB after administration of isoprenaline, suggesting NF-κB involvement in myocardial injury. Similar results were presented in several studies, which included isoprenaline-induced myocardial injury [40,41,42]. Isoprenaline induces oxidative stress in animal models of myocardial injury [15,43,44,45], which can lead to increased activation of NF-κB [38,39,45]. Pretreatment with liraglutide prevented an increase in NF-κB in isoprenaline-treated rats. This is consistent with the available data showing that liraglutide exerts anti-inflammatory effects through a decrease in NF-κB [46].

A TUNEL assay was carried out to identify apoptosis at the individual cell level. In the conducted study, the number of TUNEL-positive cells increased in isoprenaline-treated rats, and liraglutide showed its antiapoptotic effect by decreasing the number of those cells. These results were consistent with those of Lodrini et al., who showed that the number of TUNEL-positive nuclei increases after myocardial injury [47].

The current study showed an increase in BAX in isoprenaline-treated rats, suggesting increased apoptosis in the myocardium. Similar results were presented by Verma et al., who used the same experimental model of isoprenaline-induced myocardial injury [42]. There is also evidence supporting these findings, which confirmed increased expression of BAX mRNA in isoprenaline-treated rats [48]. The conducted study revealed a positive correlation between NF-κB and BAX as evidence of the involvement of NF-κB in the apoptotic pathway in experimental TTS. CC3 is crucial for the terminal phase of apoptosis [49], and the percentage of CC3-positive cells in the conducted study was increased in the myocardium of animals treated with isoprenaline. Other studies on isoprenaline-induced myocardial injury obtained similar results [41,42,48]. In addition, the results of our study showed a strong positive correlation between NF-κB and CC3. On the other hand, BCL-2, as an antiapoptotic marker, was reduced in isoprenaline-induced myocardial injury, like in other studies [42,48]. During the inhibition of apoptosis, the BCL/BAX ratio is elevated, which contributes to better cell survival [50]. Experimental studies have shown that increased expression of BCL-2 significantly reduces the size of the infarct zone in ischemia–reperfusion injury [51]. Our study showed, expectedly, a negative correlation between NF-κB and BCL-2.

Pretreatment with liraglutide revealed cardioprotective effects, particularly in terms of decreased proapoptotic and increased antiapoptotic markers. The antiapoptotic effects of liraglutide in cardiomyocytes are consistent with the results of other studies [52,53]. Interestingly, a positive correlation of NF-κB and proapoptotic markers, such as BAX and CC3, remained the same in liraglutide-pretreated rats with myocardial injury. Similarly, a negative correlation of NF-κB and BCL-2 remained the same in myocardial injury of animals pretreated with liraglutide, showing its protective effects against apoptosis. This relationship between NF-κB and proapoptotic and antiapoptotic markers in liraglutide pretreatment of experimental TTS suggests the efficiency of this GLP-1 receptor agonist in protecting the myocardium against isoprenaline-induced injury. Current information concerning the mechanisms behind the antiapoptotic effects of liraglutide in myocardial injury is insufficient. Zhu et al. [54] proposed antiapoptotic liraglutide pathway in ischemic neurons. They have suggested that liraglutide, via protein kinase B (Akt), decreases BAX and caspase-9 and -3 and increases BCL-2. On the other hand, some findings suggest that liraglutide, through Akt, inhibits NF-κB, decreases BAX and caspase-3, and increases BCL-2 in diabetic cardiomyopathy [55,56]. Further research is needed to confirm these findings in isoprenaline-induced TT-like myocardial injury.

This study has certain limitations. First, in this study, experimental TTS was induced in male rats, although in clinical practice, women are more likely to develop TTS than men [57]. However, when experimental models of TTS are concerned, both sexes can be used [4,11,58,59]; moreover, some authors demonstrated that male rats are more prone to develop experimental TTS than female rats [60]. Second, because of the excess of catecholamines in patients with TTS, adrenalin can also be used to induce TTS in animals [61]. At the same time, in animal models, isoprenaline is also used because its effects are more limited to the heart [4,12,32,58], and isoprenaline causes similar pathophysiological features as TTS [12,31]. Third, although in this experiment the apical akinesia characteristic for TTS was not demonstrated, Sachdeva et al. [11] documented it after using the same dose and the same time sequence of isoprenaline administration used in this study.

## 5. Conclusions

The current study revealed strong cardioprotective effects of liraglutide pretreatment in isoprenaline-induced TT-like myocardial injury by increasing BCL-2 and decreasing BAX, CC3, and NF-κB.

## Data Availability

The authors confirm that the data supporting the findings of this study are available within the article.
